# Conjunctival myxoid stromal tumor of the palpebral conjunctiva: A case report

**DOI:** 10.1016/j.ajoc.2022.101427

**Published:** 2022-02-18

**Authors:** Pamela S. Martin, Cooper D. Rodgers, Aldo Fantin, Gulsun Erdag

**Affiliations:** a University of Florida, 1600 SW Archer Rd, Gainesville, FL, 32608, USA; b North Florida / South Georgia Veterans Health System, 1601 SW Archer Rd., Gainesville, FL, 32608, USA

**Keywords:** Palpebral, Conjunctiva, Myxoid, Stromal, Tumor

## Abstract

**Purpose:**

To present the importance of considering conjunctival myxoid stromal tumors in the differential when evaluating eyelid lesions as these tumors could indicate undetected systemic syndromes including Zollinger-Ellison Syndrome, Carney complex, and other endocrine disorders.

**Observations:**

We present the case of a 56-year-old Caucasian female who was evaluated for a solid cyst-like structure of the palpebral conjunctiva just temporal to, but not involving, the left lower eyelid punctum. The lesion was removed with histopathologic examination of the specimen revealing the lesion to be a myxoid spindle cell tumor, consistent with conjunctival myxoid stromal tumor.

**Conclusions and Importance:**

Myxoid tumors are an abnormal proliferation of mesenchymal cells. These are most commonly found in the heart and less commonly in the bone, skin, and skeletal muscle. Myxoid tumors of the conjunctiva are a very rare reported finding and most reported cases involving the conjunctiva occur on the bulbar conjunctiva. Our patient was found to have a conjunctival myxoid stromal tumor of the palpebral conjunctiva. As these are rare lesions, we believe that considering this as a differential when evaluating eyelid margin lesions is important due to the association of these tumors with systemic conditions including Zollinger-Ellison Syndrome, Carney complex, and other Endocrine disorders.

## 1 Introduction

A myxoid tumor is a benign, abnormal proliferation of mesenchymal cells.^1^^,^^2^ These connective tissue tumors are most commonly found in the heart, skin, bone, skeletal muscle, nasal sinuses, the gastrointestinal tract, and the genitourinary system.^2^^,^^3^ Their occurrence on the conjunctiva is rare with one study reporting only twenty-eight cases between the years of 1988 and 2018.^4^ Even more rare within the subset of those occurring on the conjunctiva are those that occur on the palpebral conjunctiva as roughly 93% of previously reported cases of conjunctival myxomas were found on the bulbar conjunctiva.^2^ We present the rare case of a 56-year-old female with a slow-growing eyelid margin-involving conjunctival mass, which was identified histopathologically as a palpebral conjunctival myxoid stromal tumor.

## 2 Case presentation

**Fig. 1:**
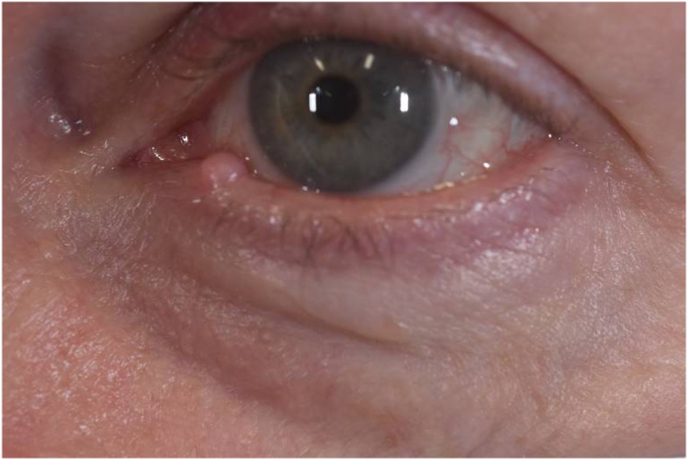

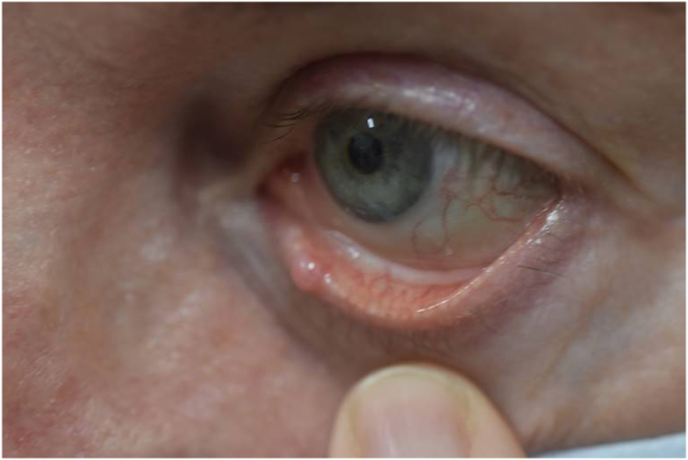
A) External photo of the left eye with eyelid lesion adjacent to the lower eyelid punctum. B) External photo of the left eye with eversion of the lower eyelid revealing lesion involvement of the palpebral conjunctiva.

Our patient is a 56-year-old year Caucasian female who was referred to our clinic for removal of an eyelid lesion. The patient stated that the lesion had been present for greater than one year and that during this time, it fluctuated in size, sometimes improving in size with warm compresses. The patient's past ocular history was notable for blepharitis and dry eye syndrome; however, she had no history of prior eye surgery. Patient's visual acuity was 20/20 in both eyes. External examination was remarkable for an elevated cyst of the left inferior eyelid margin, just temporal to the punctum with telangiectasias but no feeder blood vessels (Fig. 1). The clinical diagnosis was eyelid margin cyst.

**Fig. 2:**
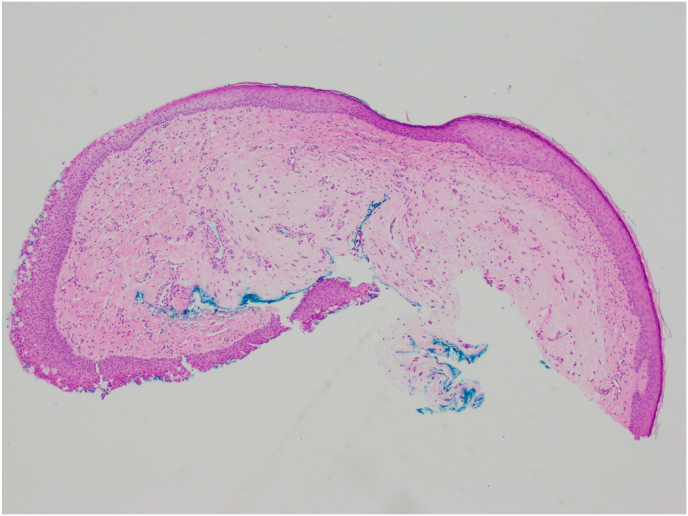

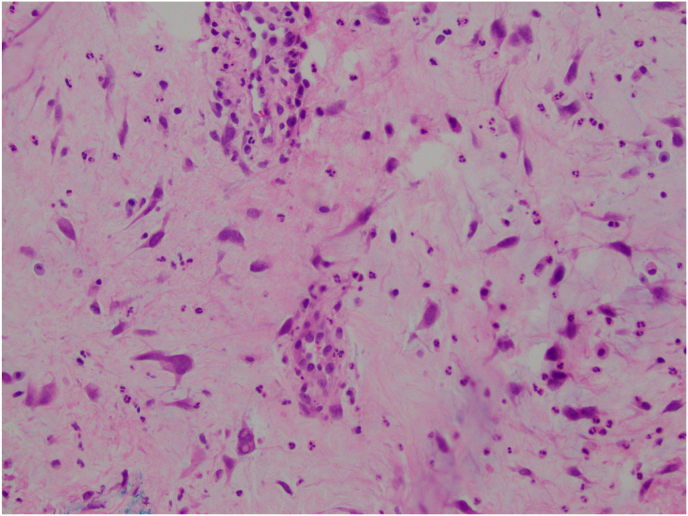
H&E stained sections showing singly dispersed spindle to round cells within a myxoid stroma. Fig. 2A) Magnification x 40. Fig. 2B) Magnification x 200. Fig. 2C). Magnification x 400 with cells showing nuclear pseudoinclusions, top arrow indicating a cell with a degenerated nucleus, bottom arrow indicating multinucleation.

**Fig. 3:**
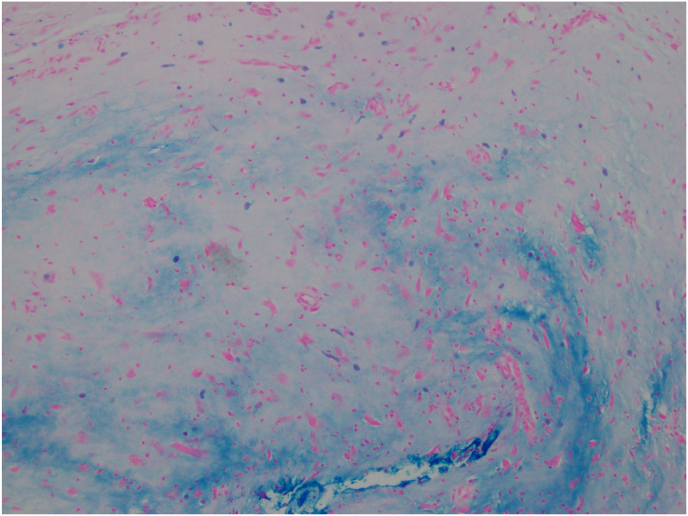

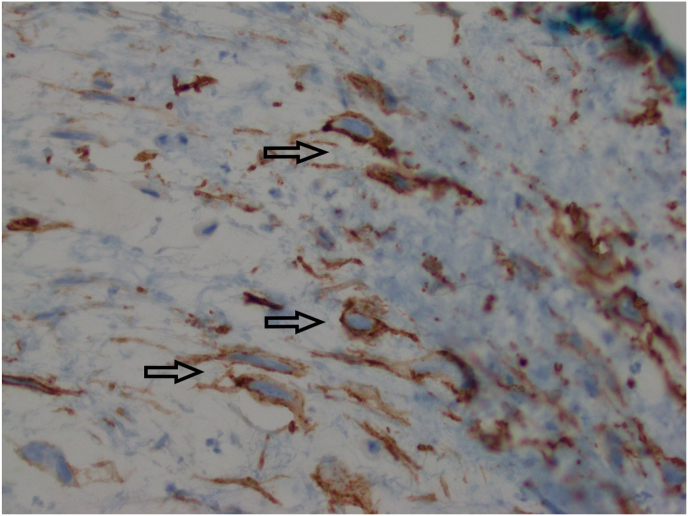
3A) Alcian Blue stain shows myxoid stroma (magnification x 100). 3B) CD34 immunostain (indicated by arrows) is expressed in the lesional cells (magnification x 400). (For interpretation of the references to colour in this figure legend, the reader is referred to the Web version of this article.)

The patient requested excision of the lesion as she was experiencing significant irritation. Due to the proximity of the lesion to the punctum, the patient was taken to the operating room for excision of the lesion. Wedge resection was considered, but ultimately not performed given the benign appearance of the lesion and the lesion location close to the lacrimal punctum. For the procedure, a 15-blade scalpel was used to perform a shave biopsy and was sent for histopathological evaluation. The tissue sample was fixed in formalin, embedded in paraffin, and 6-μm sections were obtained for routine Hematoxylin and Eosin stain and immunostaining. Histologic analysis revealed conjunctival mucosa containing proliferation of a singular spindle and round cells within a myxoid stroma (Fig. 2 A, B, C). Some of the round cells showed degenerative nuclear changes, intranuclear pseudoinclusions and multinucleation (Fig. 2C). On immunohistochemical stains, the lesional cells were positive for CD34 (Fig. 3B) and focally weakly positive for S100. They were negative for Sry-related HMg-Box gene 10 (Sox10), Cytokeratin AE1/AE3 (CK AE1/AE3), Smooth Muscle Actin (SMA), and desmin. Alcian blue staining confirmed the presence of myxoid material in the stroma (Fig. 3A). Based on morphology and immunostaining, the lesion was diagnosed to be consistent with conjunctival myxoid stromal tumor.

## 3 Discussion

A myxoma is a benign proliferation of cells of mesenchymal origin.^1^^,^^2^^,^^4^ They are most commonly found in the heart, but can also occur in the skin, bone skeletal muscle, nasal sinuses, gastrointestinal tract, and genitourinary system.^1^, ^2^, ^3^, ^4^ Myxoid lesions of the conjunctiva have been called by several names, including “conjunctival myxoma,” “conjunctival stromal tumor,” and “conjunctival myxoid stromal tumor”^4^ with Qin et al. arguing that all “conjunctival stromal tumors” and “conjunctival myxomas” be called “conjunctival myxoid stromal tumors”.^5^ Additionally, prior to the emergence of conjunctival stromal tumor as its own entity in 2012, all primary myxoid proliferations were diagnosed simply as myxomas.^4^ Myxoid tumors of the conjunctiva are a very rare entity. Milman et al. reported just 28 cases of conjunctival myxoid lesions from 1988-2018^4^ and Grossniklaus et al. reported 4 cases out of 2455 (0.002%) conjunctival lesions and Shields et al. reported 1 case out of 1643 (<0.001%) conjunctival lesions to be conjunctival myxoma.^1^^,^^2^ Histopathologically, these tumors are characterized by abundant mucoid material, a meshwork of reticulin fibers, and a small number of spindle-shaped and stellate-shaped cells.^1^^,^^3^ Immunohistochemically, myxoid tumors typically display strong positive immunoreactivity to CD34 and vimentin while having negative S100, SOX10, and SMA^4^^,^^5^ expression. The stroma stains strongly positive with Alcian blue. CD34 is a cell surface marker found on hematopoietic stem cells but is also a marker for solitary fibrous tumors. Cells of mesenchymal or endothelial origin stain positively for vimentin. S100 and SOX10 staining help to identify melanocytes as well as Schwann cells and are therefore useful in the diagnosis of melanoma and nerve sheath tumors, respectively. Lastly, SMA staining is positive in myofibroblasts.

On review of the literature, the most common location of conjunctival myxoid tumors is on the bulbar conjunctiva. Qin et al. reported on ten patients with conjunctival myxoid stromal tumors with all ten tumors occurring on the bulbar conjunctiva.^5^ Milman et al. conducted a retrospective review of all cases of conjunctiva myxoma, conjunctival stromal tumor, and reactive fibromyxoid proliferation diagnosed at Wills Eye Hospital, Emory Eye Center, and Mayo Clinic from January 1, 1988 through January 1, 2018. During this review, there were found to be just 28 patients with aforementioned lesions. 56% of lesions were localized to the limbal conjunctiva, 24% to the bulbar conjunctiva, and 4% (1 tumor) to the tarsal conjunctiva with adjacent eyelid margin involvement.^4^ It is because conjunctival myxoid stromal tumors are most commonly found on the bulbar conjunctiva that our case is important to consider. Our patient's tumor was located on the palpebral conjunctiva with involvement of the eyelid margin. With the majority of these tumors occurring on the bulbar conjunctiva, the correct diagnosis could be easily overlooked.

Oftentimes, conjunctival myxoid tumors are clinically misdiagnosed as cysts. Other differentials for these lesions include apocrine mixed tumor, nevus, amelanotic melanoma, fibrous histiocytoma, lymphangioma, myxoid neurofibroma, spindle cell lipoma, solitary fibrous tumor, rhabdomyosarcoma, pseudotumor, fibromyxoma, superficial angiomyxoma, cutaneous benign mixed tumor, and myxoid liposarcoma.^1^^,^^5^, ^6^, ^7^, ^8^, ^9^ Histopathology and immunohistochemistry are important in distinguishing these lesions. Apocrine mixed tumors display epithelial and mesenchymal components while staining positive for CD10, S100, α-SMA, and p64.^10^ Neurofibromas are tumors of neural origin and express S100. Other fibrohistiocytic tumors (fibrous histiocytoma, angiomyxoma, myxoma, solitary fibrous tumor) can be distinguished from the myxoid stromal tumor by their morphologic growth pattern and cytologic features. Additionally, CD34 is negative in these tumors. Solitary fibrous tumors express CD34, however, morphologic features (thick collagen fibers) are different from the myxoid stromal tumor. The importance of considering conjunctival myxoid tumors in the differential stems from its association with Carney complex, Zollinger Ellison Syndrome, and other systemic endocrine disorders.^1^^,^^4^^,^^11^^,^^12^ Carney complex is an Autosomal Dominant condition that is characterized by two or more of the following: mucocutaneous pigmentation, myxomas -- cardiac, mammary, or cutaneous --, endocrine hyperactivity, pituitary adenoma, unusual testicular tumors, psammomatous melanotic schwannoma.^1^ The conjunctival myxomas associated with Carney complex typically present prior to embolic events that occur secondary to cardiac myxomas, which is what makes the diagnosis of these conjunctival lesions crucial. The treatment for these lesions should be complete excision with full histopathological and immunohistochemical evaluation. With excision, reported local recurrence is rare. A workup for Carney complex should include biopsy of any suspicious skin lesions or myxomas and bloodwork testing for endocrine abnormalities, including Cushing's syndrome (serum cortisol levels), growth hormone hypersecretion (serum growth hormone levels), and thyroid disorders (thyroid stimulating hormone, free T4, T3). There are not specific surveillance guidelines for monitoring Carney complex, but the following are recommendations to perform to monitor the disease: annual echocardiogram for cardiac myxomas, annual thyroid ultrasound for thyroid nodules, annual testicular ultrasound in boys before puberty, annual measurement of 24-h urinary free cortisol excretion, annual measurement of insulin-like growth factor-1 and prolactin, and transabdominal ultrasound in women at the time of diagnosis.^13^ In addition to its association with Carney complex, conjunctival myxoid tumors can also occur in association with Zollinger-Ellison syndrome, which is caused by gastric acid hypersecretion resulting in severe acid-related peptic disease and diarrhea.^14^ The source of the gastrin production is by either a duodenal or a pancreatic neuroendocrine tumor (gastrinoma). This syndrome should be suspected in any individual with multiple or treatment-resistant peptic ulcers; peptic ulcers distal to the duodenum; peptic ulcer disease and diarrhea; enlarged gastric folds; or multiple endocrine neoplasia type 1 (MEN1).^14^ Diagnosis of this syndrome can be made by collecting fasting serum gastrin levels, which will be elevated, in the setting of a low gastric pH.

## 4 Conclusion

This case report of a conjunctival myxoid stromal tumor of the palpebral conjunctiva presents an uncommon scenario as 93% of reported conjunctival myxomas occur on the bulbar conjunctiva.^2^ Our patient's lesion was clinically diagnosed to be a cyst and was excised due to patient preference. If not for histopathologic evaluation and immunohistochemical analysis, a diagnosis of myxoid stromal tumor would have been missed. In a patient with Carney Complex, diagnosis of these lesions is of the utmost importance as the conjunctival myxoid tumors are often seen prior to the onset of ischemic events in the eye as the result of cardiac myxomas. Therefore, when evaluating conjunctival lesions, even lesions of the palpebral conjunctiva, the differential diagnosis of conjunctival myxoid stromal tumor should always be considered.

## Patient consent

Written consent to publish this case has not been obtained. This report does not contain any personal identifying information.

## Acknowledgements and disclosures

No funding or grant support

## Authorship

All authors attest that they meet the current ICMJE criteria for Authorship.

## Declaration of competing interest

The following authors have no financial interests to disclose: PSM, CDR, AF, GE.
